# Sleep Quality and Emotion Regulation Interact to Predict Anxiety in Veterans with PTSD

**DOI:** 10.1155/2018/7940832

**Published:** 2018-06-05

**Authors:** Janna Mantua, Steven M. Helms, Kris B. Weymann, Vincent F. Capaldi, Miranda M. Lim

**Affiliations:** ^1^Neuroscience & Behavior Program, University of Massachusetts, Amherst, Amherst, MA 01003, USA; ^2^Behavioral Biology Branch, Walter Reed Army Institute of Research, Silver Spring, MD 20910, USA; ^3^VA Portland Health Care System, Portland, OR 97239, USA; ^4^Oregon Health & Science University, Portland, OR 97239, USA

## Abstract

Posttraumatic stress disorder (PTSD) is a debilitating and common consequence of military service. PTSD is associated with increased incidence of mood disturbances (e.g., anxiety). Additionally, veterans with PTSD often have poor-quality sleep and poor emotion regulation ability. We sought to assess whether such sleep and emotion regulation deficits contribute to mood disturbances. In 144 veterans, using a double moderation model, we tested the relationship between PTSD and anxiety and examined whether sleep quality and emotion regulation interact to moderate this relationship. We found that PTSD predicts higher anxiety in veterans with poor and average sleep quality who utilize maladaptive emotion regulation strategies. However, there was no relationship between PTSD and anxiety in individuals with good sleep quality, regardless of emotion regulation. Similarly, there was no relationship between PTSD and anxiety in individuals with better emotion regulation, regardless of sleep quality. Results were unchanged when controlling for history of traumatic brain injury (TBI), despite the fact that those with both PTSD and TBI had the poorest emotion regulation overall. Taken together, these results suggest that good-quality sleep may be protective against poor emotion regulation in veterans with PTSD. Sleep may therefore be a target for therapeutic intervention in veterans with PTSD and heightened anxiety.

## 1. Introduction

Posttraumatic stress disorder (PTSD), which can occur after a traumatic event, is characterized by negative alterations in cognition, mood, and arousal/reactivity [1]. PTSD is prevalent in post-9/11 veteran populations; roughly 1 in 7 deployed veterans and 1 in 10 nondeployed veterans carry a diagnosis of PTSD [2]. Additionally, PTSD can have lasting negative consequences and significant disability. Compared to age- and gender-matched veterans without PTSD, veterans with PTSD have a significantly poorer functioning in a number of tested domains. For example, veterans with PTSD are less likely to be working, more likely to have physical limitations, and more likely to have committed violence in the past several years [3]. Therefore, identifying contributing factors to impairment in PTSD is critical to designing effective therapies.

Given the prevailing physiological hyperarousal present in PTSD, it is perhaps not surprising that sleep issues are common in this population. In fact, insomnia is the most commonly reported symptom of PTSD [4]. Yet, sleep issues are not simply the product of hyperarousal, as they also seem to be causal in exacerbating PTSD symptomatology. For instance, in PTSD populations, treating insomnia (via cognitive behavioral therapy) reduces PTSD symptomatology [5] and treating of obstructive sleep apnea (OSA), which is also common in PTSD, reduces symptoms as well [6]. Taken together, these results suggest that sleep quality is an important and modifiable factor that can influence PTSD severity.

Individuals with PTSD also suffer from impaired emotion regulation, which involves the voluntary or involuntary process of modulating one's own emotional experience [7]. The process of emotion regulation is implemented using a number of well-characterized cognitive techniques or strategies. These strategies are not all equally valuable. That is, some emotion regulation strategies tend to be helpful or adaptive, while others are not. For instance, “expressive suppression” (trying to hold back or hide emotions) is predictive of poor emotional outcomes (e.g., anxiety), while “cognitive reappraisal” (reframing emotional situations to make them less emotional) is predictive of healthy emotional functioning [8]. Individuals with PTSD tend to utilize more expressive suppression than cognitive reappraisal [9], and the overuse of expressive suppression may exacerbate poor psychological functioning in this population.

Importantly, the type of strategy used for emotion regulation seems to be moderated by sleep quality. Vantieghem and colleagues recently showed that individuals with sleep issues (i.e., insomnia) suppress emotions more frequently and are less likely to use cognitive reappraisal than those without insomnia [10]. A separate study showed that poorer sleep quality (e.g., higher scores on the Pittsburgh Sleep Quality Index) predicted less cognitive reappraisal use and more expressive suppression use [10]. These studies suggest that there is a potential interaction between sleep quality and emotion regulation strategy use, such that sleep quality influences emotion regulation or vice versa.

The current study therefore is aimed at examining the potential relationship between sleep quality and emotion regulation in veterans with and without PTSD. We specifically focus on a core feature of PTSD, that is, anxiety symptomatology, and examine how sleep quality and emotion regulation interact to moderate this relationship. Using a cohort of veterans with and without PTSD, we hypothesized the following: (1) Individuals with PTSD would have higher anxiety than veterans without PTSD. (2) Individuals with PTSD would have poorer sleep quality than veterans without PTSD. (3) Individuals with PTSD would utilize higher levels of expressive suppression and lower levels of cognitive reappraisal than veterans without PTSD. (4) Sleep quality and emotion regulation strategy use would interact to moderate the link between PTSD and anxiety. Furthermore, given that PTSD is often comorbid with TBI in the veteran population, we also examined the effects of TBI on these hypotheses as a contributing factor.

## 2. Materials and Methods

### 2.1. Sample

In total, 144 veterans from the Veterans Affairs (VA) Portland Healthcare System participated under a VA IRB approved protocol (MIRB number 3641, PI: Lim). Twenty-four individuals met criteria for PTSD on the basis of self-reported symptoms (cluster criteria in the PTSD Checklist 5 or PCL-5, total score > 33 [11]) (“PTSD only” group). Twenty-three individuals carried a diagnosis of TBI confirmed in the medical record (“TBI only” group). Eleven veterans met criteria for PTSD and carried a diagnosis of TBI (“PTSD + TBI” group). Eighty-six veterans without a history of trauma exposure (neither PTSD nor TBI) served as matched controls (“control” group).

### 2.2. Questionnaires

The main analyses were conducted using several self-reported questionnaires: (1) Emotion Regulation Questionnaire (ERQ) [12], (2) National Institutes of Health Patient-Reported Outcomes Measurement Information System (NIH PROMIS) anxiety items [13], and (3) Insomnia Severity Index (ISI) [14]. We also included two questionnaires for descriptive purposes: (1) Patient Health Questionnaire-9 (PHQ-9) [15] and (2) the Functional Outcomes of Sleep Questionnaire-10 (FOSQ-10) [16].

#### 2.2.1. ERQ

The ERQ is a 10-item scale that measures an individual's tendency to use specific emotion regulation strategies [12]. Two emotion regulation strategies in particular (expressive suppression and cognitive reappraisal) are assessed by this questionnaire. Individual items are 7-point Likert scales: 1 = “strongly disagree”, 4 = “neutral”, and 7 = “strongly agree”. A higher number indicates that the participant utilizes the given emotion regulation strategy more frequently.

#### 2.2.2. NIH PROMIS

The NIH PROMIS battery is a multidomain test with a range of outcomes pertaining to physical, mental, and social wellbeing. Participants were asked to rate to what extent four items about anxiety symptoms described them (e.g., “In the past 7 days, my worries overwhelmed me.”). Items are 5-point Likert scales: 5 = “never”, 4 = “rarely”, 3 = “sometimes”, 2 = “often”, and 1 = “always”. A lower number indicates higher dysfunction.

#### 2.2.3. ISI

The ISI is a 7-item measure assessing insomnia severity (i.e., difficulty initiating and staying asleep). Individual items are 5-point Likert scales: 0 = “none”, 1 = “mild”, 2 = “moderate”, 3 = “severe”, and 4 = “very severe”. A higher score indicates higher dysfunction.

#### 2.2.4. FOSQ-10

The FOSQ-10 is a 10-item measure assessing whether an individual has poor quality of life due to poor sleep quality. Individual items are 4-point Likert scales: 1 = “yes, extreme difficulty”, 2 = “yes, moderate difficulty”, 3 = “yes, a little difficulty”, and 4 = “no difficulty”; however, half of the items are a 5-point Likert scale that includes a rating of 0 = “I do not do this activity for other reasons”. The survey has 5 subscales: (1) activity level, (2) vigilance, (3) intimacy and sexual relationships, (4) general productivity, and (5) social outcomes. A lower score indicates higher dysfunction.

#### 2.2.5. PHQ-9

The PHQ-9 is a 10-item measure assessing the presence of depressive symptoms. Individual items, which enquire about how frequently participants experience the presented symptoms, are a 4-point Likert scale: 0 = “not at all”, 1 = “several days”, 2 = “more than half the days”, and 3 = “nearly every day.” A higher score indicates higher dysfunction.

### 2.3. PTSD Diagnosis

Individuals met criteria for PTSD on the basis of symptoms reported in the Posttraumatic Stress Disorder Checklist (PCL-5) [11], using a combination of cluster criteria, and total score > 33 [11]. The PTSD Checklist for DSM-5 (PCL-5) is a 20-item measure used for the screening and provisional diagnosis of PTSD, as well as assessing symptom severity. Individual items are 5-point Likert scales: 0 = “not at all”, 1 = “a little bit”, 2 = “moderately”, 3 = “quite a bit”, and 4 = “extremely”. In the current study, PTSD status was used as a categorical variable (1 = presence of PTSD, 0 = no PTSD).

### 2.4. TBI Diagnosis

Two trained researchers reviewed medical records from the Portland VA electronic medical record system to determine whether veterans had a history of TBI. Individuals with self-reported TBI that was not confirmed in the medical record were excluded from the sample.

### 2.5. Protocol

Consecutive veterans who entered the Portland VA Sleep Clinic for a clinical diagnostic sleep study were recruited to participate as part of a larger study (MIRB number 3641, factors that affect adherence to treatment for sleep apnea, PI: Lim). All participants were in the lab for overnight sleep assessments and were asked by a trained research coordinator whether they would like to participate. Veterans who wished to participate completed all questionnaires either in person, via mail, or by phone, between the time of consent and 12 months later.

### 2.6. Statistical Analyses

IBM SPSS Statistics 23 (Armonk, NY) was used to conduct statistical analyses. Analyses were conducted in several phases. First, descriptive statistical tests were conducted to determine whether groups differed at cross section in meaningful ways. One-way ANOVA tests were used to determine whether groups differed for demographic factors (e.g., age), objective sleep characteristics (e.g., TST and sleep staging), and self-reported functioning (e.g., via the FOSQ-10 and PHQ-9). If a significant difference between groups was detected, post hoc Tukey tests were used to determine which groups differed.

Next, 2 × 2 ANOVA tests (TBI versus non-TBI, PTSD versus non-PTSD) were used to assess whether groups differed for the outcome measures of interest. For this set of analyses, a 2 × 2 ANOVA was utilized rather than a one-way ANOVA so that we could examine whether PTSD and TBI interacted to synergistically affect outcomes of interest. Outcome measures were cognitive reappraisal (higher = more use of that strategy), expressive suppression (higher = more use of that strategy), ISI scores (sleep quality; higher = more sleep problems), and NIH PROMIS anxiety scores (anxiety; lower = more anxiety). In addition, an emotion regulation ratio was calculated, which consists of cognitive reappraisal divided by expressive suppression (higher = more cognitive reappraisal use, lower = more expressive suppression use), so both strategies could be assessed in one continuous outcome measure.

**Figure 1:**
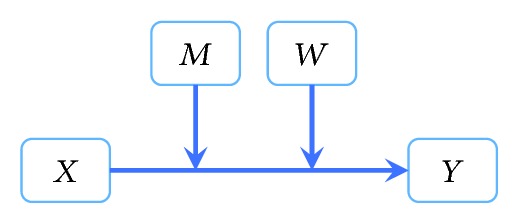
Conceptual model of the relationship between PTSD status, sleep quality, emotion regulation strategy use, and anxiety symptoms. *X* = PTSD status; *M* = sleep quality (ISI scores); *W* = emotion regulation strategy use (emotion regulation ratio); *Y* = anxiety symptoms (PROMIS anxiety items).

Lastly, given our a priori hypotheses about sleep quality interacting with emotion regulation to affect anxiety symptomatology in individuals with PTSD, moderation analyses were conducted to determine whether sleep (ISI scores) and emotion regulation (emotion regulation ratio) both moderate the relationship between PTSD (yes/no diagnosis) and anxiety (NIH PROMIS scores) (see Figure 1 for schematic). Analyses were conducted with and without TBI as a covariate to determine whether the presence of TBI impacts the relationship between PTSD, sleep, emotion regulation, and anxiety. A moderation model was chosen over a mediation model due to our a priori focus on the interaction between factors [17].

Moderation analyses, discussed directly above, were conducted using the SPSS PROCESS macro (Model 3). PROCESS calculates statistical significance using 95% confidence intervals to determine the effect of the predictor on the outcome measure. PROCESS also calculates the interaction effects of the moderators by testing the predictive effect of each factor at different levels (e.g., a low emotion regulation ratio and good sleep quality or a high emotion regulation ratio with poor sleep quality). Each predictor level was determined by values plus or minus 1 standard deviation from the centered mean. In order to eliminate both statistical power limitations and concerns about statistical assumption violations, 5000 bootstrap iterations were used within the macro (at least 2000 bootstrap iterations are recommended [18]).

Given the potential bias in our sample, which was referred for evaluation of sleep disorders, polysomnography- (PSG-) derived measures of sleep were assessed for group differences using ANOVA tests and results are reported below.

## 3. Results and Discussion

### 3.1. Preliminary Analyses

Demographic information is listed in Table 1. Notably, consistent with the demographics of veterans, the sample was predominantly male. There were no significant group differences for age, sex, or BMI between groups, although the mean age of the PTSD + TBI group was notably lower than that of the other groups. There were no group differences between objective PSG-derived sleep metrics (total sleep time (TST), sleep efficiency, sleep staging, and apnea-hypopnea index (AHI)).

**Figure 2:**
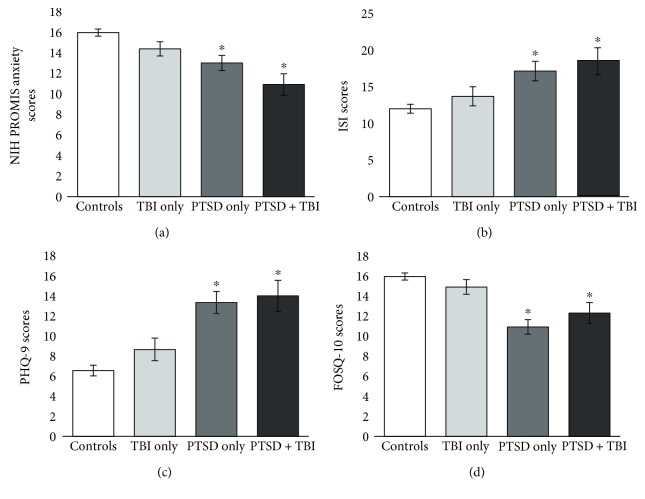
Groups differ on sleep and mental health based on TBI and PTSD status. Asterisk indicates that a given group differed significantly from veteran controls. (a) National Institutes of Health Patient-Reported Outcomes Measurement Information System (NIH PROMIS) anxiety items; (b) Insomnia Severity Index (ISI) scores; (c) Patient Health Questionnaire version 9 (PHQ-9) scores; (d) Functional Outcomes of Sleep Questionnaire version 10 (FOSQ-10) scores.

There were several mental health/quality-of-life questionnaires for which groups differed. For example, there was a significant difference between groups for the NIH PROMIS anxiety items (Figure 2(a)). Consistent with our hypotheses, post hoc tests showed that the control group had significantly lower anxiety symptomatology than the PTSD-only group (mean difference = 2.95, *p* = 0.002) and the PTSD + TBI group (mean difference = 5.05, *p* < 0.001). The TBI-only group had significantly lower anxiety than the PTSD + TBI group (mean difference = 3.46, *p* = 0.03).

Next, as hypothesized, groups differed significantly on the ISI (Figure 2(b)). Post hoc tests showed that the veteran control group had significantly lower ISI scores than the PTSD-only group (mean difference = −5.15, *p* = 0.003) and the PTSD + TBI group (mean difference = −6.49, *p* = 0.006), suggesting that subjective sleep quality was particularly poor in the PTSD and PTSD + TBI populations.

Groups also differed significantly on the PHQ-9 questionnaire (Figure 2(c)). Post hoc tests showed that the control group had significantly fewer depressive symptoms than the PTSD-only group (mean difference = 6.78, *p* < 0.001) and the PTSD + TBI group (mean difference = 7.45, *p* < 0.001). The TBI-only group also had significantly fewer depressive symptoms than the PTSD-only group (mean difference = 4.68, *p* = 0.02) and the PTSD + TBI group (mean difference = 5.35, *p* = 0.03).

Lastly, groups differed significantly on the FOSQ-10 (Figure 2(d)). Follow-up tests showed that the control group had significantly higher functioning than the PTSD-only group (mean difference = 5.03, *p* < 0.001) and the PTSD + TBI group (mean difference = 3. 64, *p* = 0.006). Additionally, the TBI-only group had significantly higher functioning than the PTSD-only group (mean difference = 3.89, *p* = 0.001).

### 3.2. Primary Analyses

**Figure 3:**
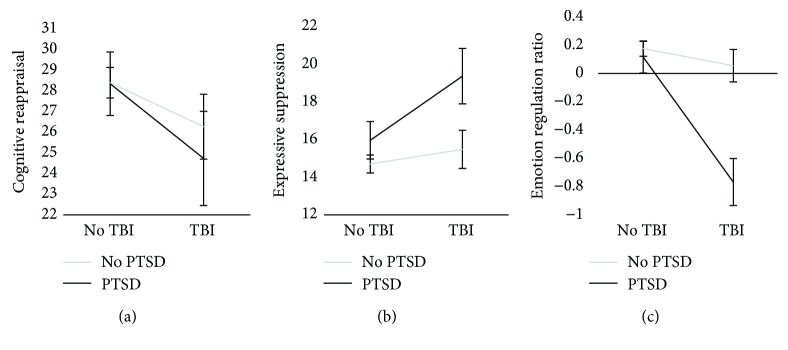
PTSD and TBI interact to predict certain outcomes. Black lines = PTSD; gray lines = no PTSD. (a) Usage of cognitive reappraisal (higher = more use of that strategy); (b) usage of expressive suppression (higher = more use of that strategy); (c) the ER ratio comprising both emotion regulation strategies (higher indicates more use of cognitive reappraisal, less use of expressive suppression).

We next conducted 2 × 2 ANOVA tests to determine whether emotion regulation strategies differed between groups. Contrary to hypotheses, when using cognitive reappraisal as an outcome measure, there was no main effect of PTSD, a trending main effect of TBI (*F*(1,158) = 3.11, *p* = 0.08; Figure 3(a)), and no interaction between the two factors. However, as predicted, for expressive suppression, there was a main effect of PTSD (*F*(1,158) = 6.04, *p* = 0.01; Figure 3(b)) and TBI (*F*(1,158) = 3.98, *p* = 0.048), such that those with PTSD and TBI used expressive suppression, a maladaptive strategy, more than controls. However, there was no interaction between these factors. Finally, the emotion regulation ratio was used as an outcome measure. There was a main effect of both PTSD (*F*(1,158) = 13.90, *p* < 0.001; Figure 3(b)) and TBI (*F*(1,158) = 17.95, *p* < 0.001), such that both the PTSD-only and TBI-only groups had a lower emotion regulation ratio than controls (indicating greater expressive suppression usage). There was also a significant interaction between the two factors, such that individuals in the PTSD + TBI group (*F*(1,158) = 10.35, *p* = 0.002) had the lowest emotion regulation ratio among the groups. Taken together, these results indicate that veterans with PTSD and TBI have an overall poorer emotion regulation profile. Additionally, PTSD and TBI cumulatively interact to create the poorest profile (i.e., the lowest emotion regulation ratio in the PTSD + TBI group).

We next conducted moderation analyses to determine the conditional effect of PTSD on anxiety symptomatology at different levels of the moderators (sleep and emotion regulation). In the omnibus model, as expected, having PTSD was a significant predictor of higher anxiety symptomatology (*B* = −1.83, 95% CI: −3.53, −0.13, *p* = 0.04). Poor sleep quality also significantly predicted higher anxiety (*B* = −1.77, 95% CI: −0.27, −0.07, *p* = 0.0005). Emotion regulation ratio significantly predicted anxiety, but in the opposite manner as predicted (*B* = −1.27, 95% CI: −2.43, −0.10, *p* = 0.03). That is, greater utilization of cognitive reappraisal (rather than expressive suppression) predicted higher anxiety. However, as described below, this main effect of emotion regulation is qualified by a significant interaction with sleep quality.

Table 2 demonstrates interactive effects between sleep quality and emotion regulation in individuals with PTSD. Notably, because these factors are moderators, a significant interaction indicates that those specific conditions must be met in order for PTSD to predict anxiety symptomatology. PTSD diagnosis significantly predicted higher anxiety in individuals who (1) utilize expressive suppression to a greater extent than cognitive reappraisal and also (2) have poor and average sleep quality. Similarly, PTSD significantly predicts higher anxiety symptomatology in individuals who (1) utilize both emotion regulation strategies equally and (2) have poor or average sleep quality. On the other hand, consistent with the above, for individuals with good sleep quality (and for individuals that predominantly utilize cognitive reappraisal, regardless of sleep quality), PTSD is not predictive of anxiety symptoms.

We conducted the same set of analyses while including TBI status as a covariate. Contrary to predictions, results were nearly identical to those noted above, indicating that the conditional effect of PTSD on anxiety symptomatology is not impacted by history of TBI.

### 3.3. Discussion

#### 3.3.1. Links between Sleep, Emotion Regulation, PTSD, and Anxiety

Our results demonstrate the strong and complex links between PTSD, anxiety, sleep quality, and emotion regulation. As expected, we found that individuals with PTSD reported more anxiety and poorer subjective sleep compared to control subjects. With regard to emotion regulation, individuals with PTSD used emotion suppression (maladaptive) more so than cognitive reappraisal (adaptive) compared to control subjects. Individuals with PTSD + TBI showed the lowest emotion regulation scores (most maladaptive) across groups.

In particular, we found that the diagnosis of PTSD predicted higher anxiety in individuals who both (1) utilize expressive suppression to a greater extent than cognitive reappraisal or utilize expressive suppression and cognitive reappraisal equally and (2) report less than good sleep quality. Thus, although anxiety was significantly correlated with poor sleep quality, there was also an interaction between sleep quality and emotion regulation strategy use. The interaction suggests that PTSD predicts anxiety symptoms *only when certain conditions are met*. That is, PTSD predicts higher anxiety in the context of different combinations of sleep quality and emotion regulation strategy use. Specifically, poor emotion regulation (i.e., greater expressive suppression use and equal utilization of expressive suppression and cognitive reappraisal) is linked with maladaptive anxiety symptoms only in the context of less than good sleep quality. When an individual is a good sleeper, the link between PTSD and anxiety is not present, regardless of emotion regulation. Therefore, having better sleep quality may be protective against poor emotion regulation.

Our findings are consistent with the results of a recent longitudinal study that examined the relationship between sleep, emotion regulation, and depression. Poor emotion regulation was found to mediate the relationship between poor sleep at baseline and depression at 6-month follow-up. The authors suggested that sleep was a causal factor in influencing emotion regulation and depression. Although our model examines anxiety and not depression, we believe that these models are not mutually exclusive, as depression and anxiety are highly comorbid [19, 20]. Thus, our findings confirm and add to a growing body of literature which suggests that emotion regulation difficulties link sleep with poor emotional health.

It is important to note that opposite causality (or bidirectional causality) is possible, as better emotion regulation could be protective against poor sleep quality. Individuals utilizing poor emotion regulation skills may have maladaptive thought processes that could disturb sleep. For instance, higher use of expressive suppression has been linked with higher levels of rumination [21], which can exacerbate sleep issues [22, 23]. Therefore, it is possible that the modification of either sleep or emotion regulation could causally reduce downstream anxiety symptomatology in veterans with PTSD.

#### 3.3.2. The Role of Comorbid TBI in Individuals with PTSD

In this study, we also aimed to assess the potentially moderating effect of traumatic brain injury (TBI) on sleep and emotion regulation. Although it is difficult to estimate cooccurrence of PTSD and TBI, a large cohort study found that one-third of veterans who had a TBI also have comorbid PTSD symptoms [24] and a separate study found that as many as 73% of individuals with TBI also had PTSD [25]. TBI and PTSD have separate but overlapping effects on psychopathology [26] and independent of PTSD; TBI has been linked with poor sleep quality [27, 28] and anxiety [29]. Therefore, when examining PTSD in a veteran population, TBI should also be considered to determine the potential influence of this factor on prolonged PTSD symptoms.

Many of the symptoms experienced during the chronic phase of recovery following TBI are similar in clinical presentation to PTSD symptoms. Specifically, anxiety, depression, irritability, and anger have been associated with both diagnoses [26]. Sleep issues are also common in both TBI and PTSD [28]. Consequently, individuals with both diagnoses tend to have a poorer functional status than individuals with only one diagnosis. For instance, veterans with both PTSD and TBI were shown to be more psychologically distressed and had poorer neurocognitive functioning than those with only PTSD or TBI [30]. Several other studies have detected neurocognitive differences between PTSD + TBI groups and TBI-only or PTSD-only groups [31, 32]. In line with these findings, for the first time, we have shown that individuals with both PTSD + TBI exhibit the most maladaptive profile of emotion regulation strategies. Individuals in the PTSD + TBI group utilize expressive suppression to the greatest extent across groups. These results indicate that the PTSD + TBI population has particularly poor emotion regulation.

However, unexpectedly, inclusion of TBI in our moderation model did not impact the outcomes of interest. In other words, the relationship between PTSD and anxiety symptomatology (and conditional the effects at each level of the moderators) was not affected by TBI history. It is possible that the effect of PTSD is “overriding” that of TBI, which is consistent with previous literature on these topics. Vanderploeg and colleagues tested a model several years ago to determine whether PTSD and TBI independently or concurrently predicted tested outcomes (e.g., cognitive and emotional symptoms) [33]. The authors of this work noted that PTSD had a much larger effect on outcomes than TBI. In accordance, our results suggest that TBI status was of little influence relative to PTSD. Another similar explanation is that PTSD alone already confers a ceiling effect on anxiety and emotion regulation, such that addition of TBI (or any other factors) will not show discernable effects.

#### 3.3.3. The Central Role of Sleep Disturbances and Anxiety in PTSD

Sleep disturbances are often thought of as a hallmark feature in individuals with PTSD. However, the impact of these sleep issues on quality of life—and the possible interrelationships between sleep and other factors—remains relatively unexplored. Our findings that sleep and emotion regulation interact to moderate the relationship between PTSD and anxiety symptoms could have meaningful implications for understanding barriers to treatment in veterans with PTSD.

For example, previous work has shown that improving poor-quality sleep improves PTSD severity. Treating sleep disorders (e.g., insomnia and OSA) lowers PTSD symptoms, such as nightmare frequency, unwanted thoughts, and distress [5, 6, 34, 35], suggesting that poor sleep actively contributes to PTSD symptomatology. Poor sleepers tend to utilize maladaptive emotion regulation strategies, potentially because poor sleep limits communication between emotion-regulating brain regions (e.g., the amygdala and the prefrontal cortex [36, 37]). Therefore, poor sleep may lead to poor emotion regulation, which could ultimately impact PTSD severity. Future work should be aimed at improving sleep and assessing whether emotion regulation also improves in individuals with PTSD.

Individuals with PTSD are 10 times more likely to have generalized anxiety disorder than those without PTSD [38]. Furthermore, veterans with both PTSD and high levels of anxiety have poorer quality of life than those with only PTSD [39, 40]. Focusing on anxiety symptomatology in PTSD could therefore provide valuable information to improve quality of life in individuals with PTSD.

#### 3.3.4. Limitations

The results of this study must be interpreted in the context of the study limitations. Our cohort was recruited from a single site from a single clinic, the Portland VA Sleep Clinic. By definition, these subjects are referred for evaluation of sleep complaints, which presents potential sample bias in our results. Thus, the sleep characteristics of our sample (and related factors) may not be generalizable to the population at large.

Similarly, our sample was predominantly male, as is generally representative of the veteran population. Because most veterans are male, we believe that our results have external validity and some generalizability to other veteran populations. However, due to this bias, we were unable to assess sex as a biological variable. Given that females tend to have more military-related mental health issues than males [41, 42], identifying poor mental health contributors in the female population is critical. Future studies should attempt to include a more diverse population.

Lastly, this study utilized a cross sectional design, limiting causal and directional interpretability. Although we surmise, based on previous literature, that sleep is causally impacting emotion regulation, it is possible that emotion regulation is impacting sleep. Poor emotion regulation itself may lead to mental health issues [12], which, in turn, could worsen sleep quality. In the absence of sampling these factors at multiple time points, any hypotheses of directionality are speculative.

#### 3.3.5. Implications and Future Directions

Given our results, there are two clear foreseeable steps that could be taken to follow up on this work. First, a longitudinal study should be conducted to determine whether sleep issues precede emotion regulation issues or vice versa. There is ample evidence to support the idea that poor sleep worsens emotional functioning, and in particular, there is evidence that poor sleep precedes the development of PTSD (but notably, PTSD does not precede the development of sleep disturbances) [43]. However, as mentioned, it is certainly possible that poor emotion regulation, which can contribute to mood disturbances, precedes poor sleep. Therefore, future work should be aimed at clarifying directionality.

After directionality is established, interventions could be implemented and assessed for efficacy. As discussed, there are many treatments for sleep disruption in individuals with PTSD, such as cognitive behavioral therapy for insomnia, image rehearsal therapy for nightmares, and medications [5, 35]. Implementing such sleep interventions may help support emotion regulation and improve anxiety symptomatology in veterans with PTSD. It is notable that subjective sleep, rather than objective sleep, was particularly poor in this population. Therefore, subjective sleep quality should be a point of intervention even in the absence of poor objective sleep. Alternatively, or perhaps concurrently, cognitive behavioral therapy focusing on emotion regulation strategies could perhaps be implemented to change emotional experience. Emotion regulation strategy modification (e.g., merely instructing individuals to use a different strategy) has been shown to be effective both experimentally [44] and therapeutically [45]. Taken together, modifying both sleep and emotion regulation could potentially lead to improved emotional outcomes, which could ultimately enhance quality of life in individuals with PTSD.

## 4. Conclusions

We found a relationship between PTSD and anxiety in veterans with poor/average sleep quality who utilize maladaptive emotion regulation strategies (i.e., utilizing expressive suppression more than cognitive reappraisal or using both strategies equally). However, in veterans who sleep well, there was no relationship between PTSD and anxiety, even when emotion regulation was poor. We posit that good-quality sleep is protective against poor emotion regulation in veterans with PTSD. Improving sleep could, in effect, improve anxiety symptomatology in this population. Future experimental work should implement sleep intervention techniques (e.g., cognitive behavioral therapy) in veterans in order to enhance wellbeing and quality of life in this group.

## Figures and Tables

**Table 1:** Demographics (mean ± standard deviation). BMI = body mass image; PHQ-9 = Patient Health Questionnaire version 9; FOSQ-10 = Functional Outcomes of Sleep Questionnaire version 10; NIH PROMIS = National Institutes of Health Patient-Reported Outcomes Measurement Information System anxiety items; TST = total sleep time; SE = sleep efficiency; NREM1 (%) = percent of the night in non-REM sleep stage 1; NREM2 (%) = percent of the night in non-REM sleep stage 2; SWS (%) = percent of the night in slow wave sleep; REM (%) = percent of the night in rapid eye movement sleep; AHI = apnea/hypopnea index.

	Controls	TBI only	PTSD only	PTSD + TBI	*F* value	*p* value
*Demographics*						
Age	59.1 ± 13.3	56.2 ± 15.3	55.7 ± 13.5	48.8 ± 21.3	1.6	0.13
BMI	33.0 ± 6.8	33.0 ± 7.5	32.2 ± 5.4	29.3 ± 5.0	1.0	0.42
Gender (% male)	89.4	90	80.1	100	2.7	0.45
*Mental health and quality of life*						
NIH PROMIS	16.0 ± 3.1	14.4 ± 4.3	13.0 ± 3.6	10.9 ± 4.0	10.6	<0.001
PHQ-9	6.6 ± 4.8	8.7 ± 4.7	13.3 ± 5.6	14.0 ± 7.1	15.0	<0.001
FOSQ-10	15.9 ± 3.1	14.9 ± 3.7	10.9 ± 3.0	12.3 ± 4.3	15.6	<0.001
ISI	12.0 ± 6.1	13.7 ± 5.04	17.2 ± 5.9	18.5 ± 5.4	6.8	<0.001
*Objective PSG-derived sleep metrics*						
TST (min)	294.3 ± 101.1	313.4 ± 67.9	311.2 ± 89.0	286.4 ± 113.5	0.4	0.76
SE (%)	69.0 ± 18.7	72.9 ± 13.9	71.5 ± 18.6	65.4 ± 22.7	0.5	0.69
NREM1 (%)	12.1 ± 5.7	12.7 ± 4.9	11.6 ± 5.4	10.1 ± 3.8	0.6	0.65
NREM2 (%)	42.5 ± 15.7	46.1 ± 15.3	46.2 ± 13.7	38.7 ± 20.3	0.8	0.49
SWS (%)	1.7 ± 4.2	1.2 ± 3.1	1.3 ± 3.8	1.5 ± 2.5	0.1	0.94
REM (%)	11.7 ± 7.4	11.8 ± 6.9	11.0 ± 5.7	13.7 ± 9.5	0.3	0.81
AHI	19.5 ± 20.0	13.7 ± 11.1	16.5 ± 11.7	18.5 ± 15.5	0.7	0.57

**Table 2:** PTSD presence predicts higher anxiety symptoms at each level of the moderators. Bold indicates statistical significance.

Emotion regulation strategy	Sleep quality	Beta	SE of beta	*t*	*p*	Lower CI	Upper CI
Suppression	Good	−2.26	1.56	−1.44	0.15	−5.36	0.83
Suppression	**Average**	**−2.31**	**1.09**	**−2.11**	**0.03**	**−4.48**	**−0.15**
Suppression	**Poor**	**−2.37**	**1.06**	**−2.23**	**0.02**	**−4.47**	**−0.27**
Equal utilization	Good	−1.77	1.40	−1.26	0.20	−4.55	1.00
Equal utilization	**Average**	**−1.82**	**0.85**	**−2.12**	**0.03**	**−3.52**	**−0.12**
Equal utilization	**Poor**	**−1.88**	**0.82**	**−2.27**	**0.02**	**−3.51**	**−0.24**
Reappraisal	Good	−1.28	1.47	−0.87	0.38	−4.19	1.62
Reappraisal	Average	−1.33	0.97	−1.37	0.17	−3.26	0.58
Reappraisal	Poor	−1.39	0.95	−1.45	0.14	−3.27	0.49

## Data Availability

The final, complete dataset will be available to interested users under a VA-approved data-sharing agreement that provides for: (1) a commitment to using the data only for research purposes and not to identify any individual participant; (2) a commitment to securing the data using appropriate computer technology; and (3) a commitment to destroying or returning the data after analyses are completed. Because there remains the possibility of deductive disclosure of subjects with unusual characteristics, data disclosure will be considered on a case-by-case basis; interested users may contact the corresponding author directly to initiate this process.
